# AI-Driven Analysis of Neglect in Dementia Caregiving: Insights from Unstructured Clinical Notes

**DOI:** 10.1093/geroni/igaf122.2239

**Published:** 2025-12-31

**Authors:** Jessica Hernandez-Chilatra, Carolyn Pickering, Xiaoqian Jiang

**Affiliations:** UTHealth Houston, Houston, Texas, United States; UTHealth Houston, Houston, Texas, United States; UTHealth Houston, Houston, Texas, United States

## Abstract

Understanding neglect in the dementia caregiving context requires more than detection; it demands insight into caregiving scenarios, risk factors, and potential preventive strategies. Clinical notes contain rich, unstructured data, yet traditional methods fail to efficiently extract patterns that can inform caregiving prevention interventions. This study leverages AI-driven natural language processing (NLP) techniques to analyze dementia patient clinical notes, uncovering key caregiving challenges, neglect risks, and early intervention opportunities. We analyzed 379 clinical notes from 18 dementia patients, applying MPNet embeddings to assess similarity and detect highly redundant notes. Cosine similarity scores identified 1,146 highly similar notes, which were then grouped at the patient level. GPT-4 was subsequently used to extract structured neglect insights, categorizing risks into medical, emotional, and financial domains while providing caregiving recommendations. A general summary was generated to identify common neglect patterns, risk factors, and potential interventions. Recurrent neglect patterns included unmanaged pain, dehydration, and medication nonadherence; social isolation and unaddressed distress behaviors; and missed healthcare due to financial strain. Caregiver burnout, gaps in follow-up care, and lack of support were common challenges. GPT analysis synthesized high-risk neglect patterns, anonymized case examples, and targeted interventions to inform prevention strategies. A final structured report highlighted high-risk neglect patterns, real-life examples (anonymized quotes), and suggested care interventions. This study demonstrates how AI-driven text analysis can provide actionable insights for identifying and mitigating dementia caregiving neglect. Future work may explore risk-scoring models using embeddings and structured insights to support early detection and intervention strategies in clinical practice.

